# 3D modeling and reconstruction of plants and trees: A cross-cutting review across computer graphics, vision, and plant phenotyping

**DOI:** 10.1270/jsbbs.21074

**Published:** 2022-02-03

**Authors:** Fumio Okura

**Affiliations:** 1 Graduate School of Information Science and Technology, Osaka University, 1-5 Yamadaoka, Suita, Osaka 565-0871, Japan

**Keywords:** 3D reconstruction, 3D modeling, plant phenotyping, computer vision, computer graphics, FSPM

## Abstract

This paper reviews the past and current trends of three-dimensional (3D) modeling and reconstruction of plants and trees. These topics have been studied in multiple research fields, including computer vision, graphics, plant phenotyping, and forestry. This paper, therefore, provides a cross-cutting review. Representations of plant shape and structure are first summarized, where every method for plant modeling and reconstruction is based on a shape/structure representation. The methods were then categorized into 1) creating non-existent plants (*modeling*) and 2) creating models from real-world plants (*reconstruction*). This paper also discusses the limitations of current methods and possible future directions.

## Introduction

The structure of plant shoots (i.e., leaves and stems) is an important cue for plant phenotyping and cultivation. Although modeling plant stems are known to be beneficial, it involves a number of technical challenges. To automatically *model* non-existent virtual plants, sophisticated representations of plant structure and shape are required. If we want to *reconstruct* the plant shape/structure based on observation, such as multiview images, difficulty arises because of heavy occlusions or structural complexity.

Three-dimensional (3D) modeling of plants and trees has been developed in multiple research fields. Recently, applications for plant science, breeding, and cultivation have been actively developed in the plant phenotyping (PP) field, while technical components of 3D modeling have been primarily related to the computer vision (CV) field. As so, a series of workshops named Computer Vision Problems in Plant Phenotyping and Agriculture (CVPPA), has been held in conjunction with major CV conferences. In addition, there has been an important demand for (semi-)automatic plant modeling in computer graphics (CG) because modeling plants and trees is time-consuming. Tree modeling and reconstruction are also essential topics for forestry studies analyzing forest inventories.

In this paper, a cross-cutting review of the 3D modeling methods of plant stems is presented, spanning across research fields such as CV, CG, and PP. There has been some survey literature related to 3D modeling of plants. For instance, a recent survey paper has discussed 3D reconstruction for plant phenotyping (Paulus 2019), including a brief theory of 3D reconstruction. A more specific topic, multiview image-based plant modeling, was summarized in (Kochi *et al.* 2021). The present paper aims to provide a broader and cross-cutting review, including the state-of-the-art from multiple research fields. Although it only focuses on plant stems (i.e., the above-ground part), the root system is also an essential target of plant modeling (e.g., Zheng *et al.* 2011) and interested readers are encouraged to refer to a recent survey report of root phenotyping (Takahashi and Pradal 2021). Image-based plant phenotyping has been well studied for related topics (Li *et al.* 2014, 2020b). More specifically, unmanned aerial vehicle (UAV)-based phenotyping (Guo *et al.* 2021) and the use of convolutional neural networks (CNNs) for plant phenotyping (Jiang and Li 2020, Toda and Okura 2019) are also summarized. A specific review of the 3D representation of plant structure/architecture has been presented earlier (Godin 2000).

## Overview

First, a brief classification of plant modeling/reconstruction studies is summarized. For simplicity, the following definitions of the terms *modeling* and *reconstruction* are used in this paper:

• Modeling: Creating models of non-existent plants by simulating their shapes and structures;

• Reconstruction: Creating plant shapes or structures which mimics existing plants.

Note that *modeling* is generally used for broader meanings, including reconstruction tasks (e.g., *image-based modeling* is regarded as a reconstruction task).

In addition to the methodologies for modeling and reconstruction, the representation of plant shape and structure is an important topic of this study. While we can naturally represent the plant shape using point clouds and mesh models, structural representations are often used for (functional) structural plant modeling ((F)SPM), which are frequently used for the simulation of plant functionality (Kim *et al.* 2020).

**Fig. 1.**
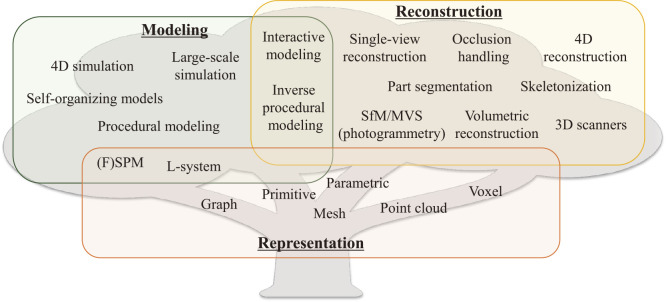
A rough classification of important words used in plant modeling/reconstruction techniques.

Fig. 1 summarizes the classification of plant modeling/reconstruction. In this paper, the major representations of plant shape and structure are first summarized, and a review of plant modeling/reconstruction methods using these representations is then presented.

## Plant shape/structure representation

**Fig. 2.**
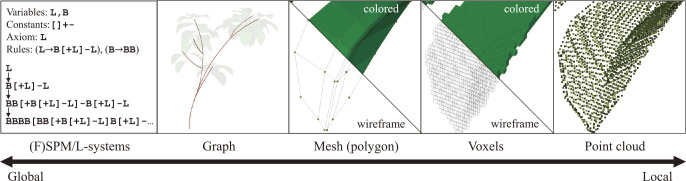
Shape and structure representations of plants. The left-most column shows an example of structural representation, L-system, which generates structural patterns via recursive processes (see Fig. 3 for details).

This section briefly reviews on how 3D shapes and structures are represented in the virtual world. Both plant-specific approaches and general 3D representations are introduced. Fig. 2 summarizes the common representations of local and global shapes/structures.

### Shape representations

There are multiple representations for some shape details. Local representations are first described, followed by ways to represent the shape and structure globally.

#### Point cloud representation 

A straightforward way to represent an object’s existence at a specific 3D location is to use a point or density plot. Point-based 3D representations are often called point clouds consisting of 3D points located on the surface of an object. As a default, many commercial range scanners, light detection and ranging (LiDAR), and depth sensors yield point clouds as 3D measurements. Many plant reconstruction methods use point cloud input given by multiview stereo or 3D laser scanners.

#### Voxel representation 

Volumetric representations are commonly used in density/silhouette-based 3D reconstruction methods, such as computed tomography (CT) (Brooks and di Chiro 1975), which represent the object density of each small 3D grid (i.e., voxels). Interested readers are invited to refer to the fundamental techniques in CG (Foley *et al.* 1996). An early attempt at the 3D reconstruction of botanical trees used a voxel-based representation for the crown (Reche-Martinez *et al.* 2004), which enabled practical CG applications such as relighting, i.e., simulating tree appearances under different illumination conditions (Cabral *et al.* 2011). In addition, X-ray CT is a major method for analyzing the 3D shape of grains (Hu *et al.* 2020, Hughes *et al.* 2017).

#### Mesh (polygon) representations 

Gathering the local shape is often helpful beyond just the point or density at a 3D position. A common way of representing 3D shapes by local shapes is to use small planes, i.e., polygon meshes. An advantage of mesh-based representation is the simplicity of deriving the neighboring points, resulting in a simple computation for the surface normal direction or object boundary, which are essential when using 3D models for rendering or physics simulation. Converting point/voxel-based representations to polygon meshes is useful in terms of frequency. For example, Poisson mesh reconstruction (Kazhdan *et al.* 2006) generates a mesh model from a given point cloud. From voxel representations, we can use an intermediate representation such as a signed distance function (SDF) (Curless and Levoy 1996) during the conversion, which represents the distance from the object surface (and the zero-crossing indicates the surface location). However, obtaining reasonable meshes from a point cloud or voxels is often challenging for thin objects such as plant stems. Recent studies have attempted to overcome this issue using deep learning and have shown promising results, including plant 3D models (Wei *et al.* 2021).

#### Parametric surface representations 

Parametric (curved) surface representations, such as Bezier, B-spline, and NURBS (Piegl and Tiller 1997), are used to represent more global shapes using a smaller number of parameters than the polygon models. For example, leaf shapes can be approximated using a curved surface. Some methods for leaf 3D reconstruction fit the parametric surfaces on the given observation in the form of point clouds (Ando *et al.* 2021, Quan *et al.* 2006).

#### Primitive-based representations 

If we have prior knowledge of the target scene, we can interpret it as a composition of primitive shapes, such as cylinders. For example, early studies on human image analysis frequently approximated human shapes using multiple cylinders (called cylindrical models) (Deutscher and Reid 2005). Indoor scenes or city sceneries are often approximated by the composition of cuboid shapes (called the Manhattan World assumption (Coughlan and Yuille 1999)). In the context of plant modeling, cylindrical models are sometimes used because the branches can be approximated as stacks of small cylinders (Tan *et al.* 2007, 2008).

#### Neural implicit representations 

Recent advances in neural networks enable the representation of 3D shapes as weight parameters in a neural network, called neural implicit representations. Neural radiance fields (NeRF) (Mildenhall *et al.* 2020) are a typical example based on a ray-based representation. The density and color of each 3D location are *implicitly* encoded as a neural-net-based mapping function that inputs a viewing ray and a given 3D location. Similar ideas are used to represent voxel density (Niemeyer *et al.* 2020) or surface meshes (Zhang *et al.* 2021b). These representations are difficult to interpret by humans, but they demonstrate the visually promising performance of 3D (or 2.5D) shape reconstruction for thin objects, including plants.

### Structure representations

Because the structure (e.g., of branches) is essential to represent plants, 3D shapes are often converted to structure-based representations. Apart from plants, structure-based representations for human image analysis have been well studied in CV. The human pose is described by a skeleton, which is a graph structure with a fixed number of joints and fixed edge connections (i.e., isomorphic graphs). Leveraging this characteristic, the estimation of human skeletal pose has been well studied (Cao *et al.* 2021). The estimated pose can be effectively used in many applications, such as action recognition (Ke *et al.* 2017, Yan *et al.* 2018). Beyond the skeleton, the rough 3D shape of humans, including their physique, is approximated using a small number of (<100) parameters (Loper *et al.* 2015). These parametric shape representations are useful for simultaneously estimating pose and shape (Bogo *et al.* 2016, Pavlakos *et al.* 2018). These types of representations are not limited to humans; extensions to animals, for example, are available for both skeletal pose representation (Mathis *et al.* 2018) and parametric shape representation (Zuffi *et al.* 2017, 2018, 2019).

#### Graph-based representation 

For plant modeling purposes, compared to humans and animals, there is a technical challenge, because the number of joints and topology is different for each individual plant. A straightforward approach to representing plant structure is to use graph theory. Many plant modeling methods implicitly or explicitly assume that plants and botanical trees form the tree structure. For example, some (botanical) tree reconstruction methods from multiview images track the branch patterns from the root detected from a given image (Tan *et al.* 2007). The resulting structure will naturally be the tree structure in this case. Also, graph/tree-based structure is applicable for many other parts in plants, such as leaf vein, which is used for leaf species classification (Yu *et al.* 2020).

#### (Functional-)structural plant modeling (FSPM) 

**Fig. 3.**
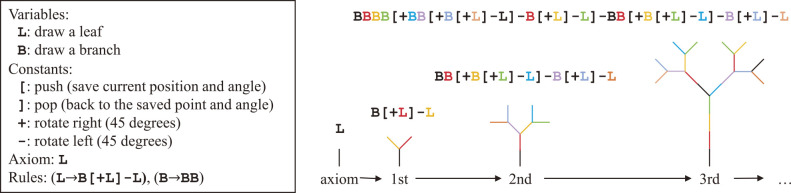
A simple example of L-system representation (binary trees). Left: Pre-defined rules. Right: Growth via a recursive process (colors of the line segments corresponding to those in the symbols).

Beyond the graph structure, description of the growth of plant structure has been well studied since early 1970’s (Horn 1971). Therefore, it is natural to use such characteristics for plant modeling purposes. One of the important classes of plant structure representation is FSPM (Vos *et al.* 2010), which unifies the properties of plant growth in the model representation. This class of representation has been studied for a long time, even before naming it FSPM, as presented in (Honda 1971). A famous example of FSPM-type representation is the Lindenmayer system (L-System) (Lindenmayer 1968, Prusinkiewicz *et al.* 1994), which is a formal language used to describe the growth (evolution) of the structural shape. It includes production rules, which define how to replace the symbols. Fig. 3 illustrates a simple example of the growth with the L-system, which generates the binary trees. Based on the pre-defined rules, we get strings via recursive processes. The strings can be decoded into structures using the definitions of variables and constants. For details on L-systems, interested readers can refer to Prusinkiewicz and Lindenmayer (1990). A possible extension is to introduce self-organization in plant growth, where each *unit* (e.g., a branch) in a plant decides how to grow (or die), considering the surrounding environment (Palubicki *et al.* 2009, Ulam 1962).

FSPM has been actively studied and implemented. L-studio (Prusinkiewicz *et al.* 1999) is an early implementation of an L-system-based simulator. An extension of the L-system, relational growth grammars (RGG), (Kniemeyer *et al.* 2007, Kurth *et al.* 2004) and the programming language XL (Hemmerling *et al.* 2008) are used in the software GroIMP (Kniemeyer *et al.* 2006). Another famous project is OpenAlea (Pradal *et al.* 2008), which develops an integrated environment using Python, including the visualization library PlantGL (Pradal *et al.* 2009). In addition, many commercial applications now implement structure-based representations, such as Xfrog (Deussen and Lintermann 2005) and SpeedTree (https://speedtree.com/). Previous literature describes how each plant component has been parameterized so far (Prusinkiewicz and Runions 2012).

#### Representations for effectiveness 

Another research direction on the representation of trees and plants has been how to improve its efficiency. The hierarchical structure of trees expresses abstract shapes of foliage details by canonical geometry (named texture-lobes), resulting in lighter but still plausible 3D models (Livny *et al.* 2011) for efficient transmission or light-weight simulation. A similar direction was introduced in (Quigley *et al.* 2018), representing trees with a limited number of rigid bodies.

## Modeling of (virtual) plant shape/structure/appearance

A major goal of generating plants and trees in the synthetic environment is the automatic setup of synthetic environments, which is useful for plant phenomics or photorealistic CG simulations (e.g., for games and cinemas). Because it is time-consuming to create hand-crafted CG models of plants and trees, (semi-)automatic modeling is well studied as part of the FSPM study and CG community.

**Fig. 4.**
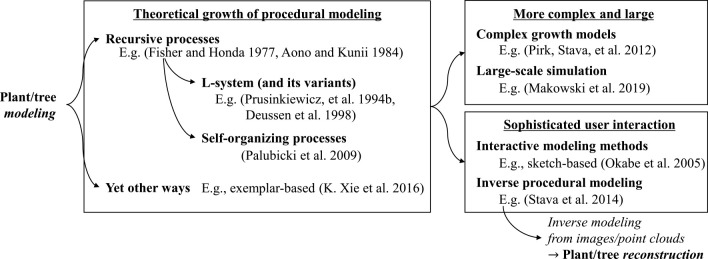
Developmental trends in plant modeling methods. The theoretical growth of procedural modeling (left) has been developed to the more complex and large-scale approaches (top right) or sophisticated through user interaction (bottom right).

The technical components are closely related to the structure-based representation of the plants, such as the L-system. In contrast, to create plausible plant shapes and structures, it is mandatory to analyze how to determine the production rules for structure-based representations. Plant modeling is often categorized into a *procedural modeling* pipeline based on a *growing* procedure, which is also used for the generation of terrain, water surface, and city layouts (see Smelik *et al.* (2014) for a recent review). So far, many studies have considered the interaction during growth within the same plant, with the surrounding environment, or manipulation by users. Fig. 4 summarizes existing studies on plant/tree modeling.

### Procedural modeling of plants and trees

The evolution of procedural modeling pipelines for the modeling of plants and trees is presented next.

#### Early works with recursive processes 

For the modeling of botanical trees, Honda and colleagues provided some examples (Fisher and Honda 1977, Honda *et al.* 1981) based on the analyses of the branching angles and lengths (Honda and Fisher 1978, 1979) using the representation of tree structures in a recursive manner (Honda 1971). In addition, in CG literature, early work on the generation of 3D shapes of trees was based on fractal-based recursive algorithms to create artificial plants and trees (Aono and Kunii 1984, Oppenheimer 1986). These were localized to model specific species (Bloomenthal 1985), extended to real-time modeling (Oppenheimer 1986), or used to discuss how they could be rendered realistic (Reeves and Blau 1985). In addition, de Reffye *et al.* (1988) tried to convey detailed botanical knowledge in the recursive process, such as how they generate branches, occupy spaces, or create leaves/flowers.

#### Using the L-system (or its variants) 

Using the L-system representation or its variants is a common way of modeling plant structure. A simple method is to generate a plant/tree using the L-system and then prune the branches to fit a given volume or silhouette (Prusinkiewicz *et al.* 1994, Weber and Penn 1995). Further extending L-systems has been studied to consider environmental effects, such as collisions, space competition (for colonization or growth), and competition for light and water (Měch and Prusinkiewicz 1996). Early reviews in this direction can be found in Prusinkiewicz (1998) and Prusinkiewicz *et al.* (1997). Deussen *et al.* (1998) introduced an overall procedural modeling pipeline from terrain generation, ecosystems, geometric plant models, and other given components. In addition to the related literature by Lintermann and Deussen (1998, 1999), a famous Xfrog modeling system was developed. To represent more branch-level details, Streit *et al.* (2005) attempted to model the detailed shape (e.g., the curvature of a branch), which is often beyond L-system-like representations. In addition, Galbraith *et al.* (2004) aimed to represent detailed texturing effects, such as branch bark ridges and bud scale scars. To represent the space competition among branches and leaves, the space colonization technique limits the space in which each branch can grow (Runions *et al.* 2005, 2007).

#### Self-organizing processes 

It is relatively easy to prune the branches to fit the given silhouettes or volumes using L-system-like representations. However, it is not straightforward to control the behavior of each element (e.g., bud and branches), resulting in unrealistic models. A promising approach to this problem is to introduce the idea of a *self-organizing* process, where each element considers how it will grow according to the surrounding environment, for example, to maximize the space for each element (Ulam 1962). Self-organizing tree modeling (Palubicki *et al.* 2009) is a cornerstone for this direction, which can incorporate the effect of surroundings such as space colonization or shadows (or additional user input) to determine the fate of each bud. This characteristic enables users to generate highly realistic tree models with simple and easy interactions.

### Recent direction of procedural plant/tree modeling

Recent studies have attempted to represent more complex or large environments based on procedural modeling methods.

#### Complex growth models 

The recent growth of computing resources enables us to use rich information during procedural modeling. For example, Wang *et al.* (2014a) evaluated the cost function every time a branch was added. The cost function assesses the fitness of the crown shape with the given silhouettes and other botanical priors. Yi *et al.* (2018) introduced diverse factors (e.g., detailed lighting and occupying spaces) into a growth equation and evaluated them during the procedural modeling simulation.

#### Complex environmental interaction 

Plastic Trees (Pirk *et al.* 2012a) considers the dynamic deformation of trees by environmental interactions, such as the occurrence of new objects colliding with the growing tree. Wind effects during growth are also discussed (Pirk *et al.* 2014), where they consider the wind force, branch breaking, bud abrasion, and drying. The interaction with supporting objects for climbing plants was discussed in (Hädrich *et al.* 2017).

#### Large-scale simulation 

While there have been attempts to make large-scale scenery simulations (Beneš *et al.* 2009), fast or real-time simulation has been performed at the forest level (Eloy *et al.* 2017, Kim and Cho 2012, Makowski *et al.* 2019).

#### Other methods or applications 

There have been tree/plant modeling methods other than the recursive/self-organizing processes. One of these methods sets up tree structures to connect the randomly distributed points by graph optimization and manual interaction, resulting in trees with irregular appearance representing some environmental effects (Xu and Mould 2012). Similarly, the growth direction can be controlled in more detail by setting a vector field in 3D space, where the branches grow along the vectors (Xu and Mould 2015). The exemplar-based method was proposed in (Xie *et al.* 2016), where real tree parts were combined to represent tree models. Along this line, blending *between* trees in the shape space was proposed (Wang *et al.* 2018a), which can generate time-series tree models. The methods studied for plant/tree modeling are applied to other (yet similar) applications, such as creating opening flowers (Ijiri *et al.* 2008).

### User interaction in plant and tree modeling

Beyond generating the models that fit given silhouettes and volumes, sophisticated methods for interactive modeling have been proposed. For example, Boudon *et al.* (2003) focused on user interaction via a graphical user interface (GUI) to create Bonsai trees. A common way to efficiently create 3D models is to use sketches/scribbles. Sketch-based interactions to create tree models have been well studied (Ijiri *et al.* 2006a, Okabe *et al.* 2005) and extended to the modeling of flowers (Ijiri *et al.* 2005, 2006b). Inferring a branching structure from rough concept sketches (Anastacio *et al.* 2006) is along this line, and some methods further optimized multiple shape parameters from sketches (Anastacio *et al.* 2009, Chen *et al.* 2008) or silhouettes (Wither *et al.* 2009). Decreasing the required number of sketches is a promising direction (Longay *et al.* 2012) and distinguishing the type of sketches, e.g., sketch for main branches and spray for foliage (Zakaria and Shukri 2007), has also been studied. Another unique direction (Onishi *et al.* 2003, 2006, Zhang *et al.* 2021a) is to develop a user interface for manipulating tree models in a virtual reality space. A recent method directly infers L-systems from line drawings (Guo *et al.* 2020a), which shows a potential of using deep learning for the estimation of structural representations.

### Inverse procedural modeling

Another possibility of user-guided plant modeling is to provide richer cues (than sketches or silhouettes) to the modeling system, namely, to provide photographs of plants/trees or an existing 3D model (e.g., polygon meshes or a point cloud) to infer plant/tree structures. This batch of methods is sometimes called *inverse* procedural modeling (Stava *et al.* 2014), which is also regarded as a reconstruction problem. The next section will further revise this problem, while here, the review will focus on how these methods can be used for plant/tree simulation.

Some may recover the plant/tree structure from the existing 3D polygon model created by Xfrog. For example, using a polygon model, Pirk *et al.* (2012b) estimated the skeleton structure and then created a backward growth animation from the given model. In addition to the skeleton structure, polygon models can be converted into a hierarchical structure associated with the original meshes, enabling physics simulation (Zhao and Barbič 2013).

Extracting 3D structures from existing real-world plants is a quick way to model virtual plants. Many approaches have been studied to achieve this goal by inputting a point cloud acquired by 3D scanners (Livny *et al.* 2010, Xu *et al.* 2007), multiview images (Isokane *et al.* 2018, Neubert *et al.* 2007, Tan *et al.* 2007, Wu *et al.* 2020), or even a single image (Argudo *et al.* 2016, Tan *et al.* 2008). These can be used for growth simulations or graphical applications, as shown so far. However, it should be noted that the goal of reconstruction is different based on its applications. For example, because the goal of graphical applications is to create 3D models with plausible and photorealistic appearance, some methods are not suitable for direct use of plant breeding-related applications that require 3D models faithful to real objects.

## Reconstruction of plant shape and structure

A straightforward way to create a 3D plant model from real-world plants is to peel each leaf off and measure it using 2D/3D scanners (Yin *et al.* 2016); however, this is unrealistic for many cases, for example, growth analysis. Therefore, many attempts have been made to reconstruct the shape and structure of non-inversive plants from 3D point clouds or photographs. Common and general methods to reconstruct 3D shapes are first presented, followed by the methods for extracting plant structures.

### Reconstruction of 3D shapes

To reconstruct the structure of real-world plants, we first need to acquire the shape of the target scenes, such as using 3D laser scanners or multiview images. This has been considered a fundamental problem in CV for a long time. Therefore, reconstruction methods include several approaches. Technical details are not all presented but a summary of the input and output of some common techniques is given in Table 1. For technical details, interested readers are invited to read (Szeliski 2011), while a few survey papers (Kochi *et al.* 2021, Paulus 2019) provide brief overviews of plant reconstruction using these techniques.

In Table 1, the practical settings for these methods are categorized into passive and active. Active settings rely on external light sources whose positions and directions are known. Regarding the underlying approaches for each method, geometric methods use triangulation or 3D ray intersections and usually output depth images, 3D point clouds, or mesh models. Photometric methods analyze the irradiance values captured by cameras, resulting in the estimation of surface normals. In practice, it is important to know that these methods do not yield the absolute scale of the resultant models unless we place reference objects with known sizes such as ground control points (GCPs) or fiducial markers. Conversely, 3D laser scanners and the LiDAR approach measure the traveling distance of emitted light via phase differences, which output 3D shapes with an absolute scale. We then summarize the advantages and disadvantages of each approach for 3D reconstruction of plant shapes.

#### Shape reconstruction using 3D scanners 

For the reconstruction of plants and trees, 3D scanners are typically used. We can directly acquire (relatively) accurate 3D point clouds; thus, there are numerous ways of extracting the 3D structure of plants and trees from point clouds. However, there are some drawbacks to the direct use of 3D point clouds acquired by 3D laser scanners. The use of accurate laser scanners is not realistic for some applications in terms of costs or physical limitations (e.g., difficulties in mounting on drones or capturing from multiple views). In addition, 3D point clouds lack spatial relationships among the points. We usually need to be concerned about which points are physically neighboring, which is a fundamental cause of 3D point cloud processing being much more challenging than 2D image input for CV-related methods such as semantic segmentation.

#### Multiview 3D reconstruction with photogrammetry 

Another common approach is 3D reconstruction from multiview images. Structure-from-motion (SfM) is used to estimate camera poses and a sparse point cloud. Multiview stereo (MVS) was then used to estimate the dense surface shape from the given camera poses. This pipeline is also called *photogrammetry*. Using multiview images easily leverages the rapid growth of computer vision techniques, such as 2D/multiview image processing and image-based 3D reconstruction. Recent studies have provided sophisticated open-source photogrammetry implementations, such as COLMAP (Schönberger and Frahm 2016, Schönberger *et al.* 2016). Commercial photogrammetry software such as Metashape (https://www.agisoft.com), 3DF Zephyr (https://www.3dflow.net/), and RealityCapture (https://www.capturingreality.com), include useful features such as the automatic recognition of fiducial markers. Meanwhile, photogrammetry of plants and trees is sometimes challenging because of the repetition of similar textures, resulting in a low-quality outcome or failure of 3D reconstruction. Therefore, to achieve a better and faster reconstruction of 3D models for high-throughput phenotyping, some studies have discussed ways to develop multiview imaging systems (Gao *et al.* 2021, Tanabata *et al.* 2018, Wu *et al.* 2020), or the ways to select suitable images from multiple images (Lou *et al.* 2014). Wang *et al.* (2018b) provided a comparison of 3D laser scanning and MVS for plant shape reconstruction.

#### Volumetric 3D reconstruction 

As a similar setting but different method, the use of cameras surrounding a plant or tree enables volumetric approaches like shape-from-silhouette methods, which have been used for tree shape reconstruction (Phattaralerphong and Sinoquet 2005, Reche-Martinez *et al.* 2004, Shlyakhter *et al.* 2001) and phenotyping systems (das Choudhury *et al.* 2020). While the resolution of the resultant 3D shape by the naïve methods for volumetric reconstruction is capped by the voxel resolution, Klodt and Cremers (Klodt and Cremers 2014) proposed an optimization framework to acquire the volumetric reconstruction of plants with fine details by optimization using octrees. X-ray CT shares theoretically similar ideas to these volumetric approaches, and it is used for plant reconstruction (Ijiri *et al.* 2014).

#### Photometric methods for 3D reconstruction 

Compared to geometric approaches such as MVS and shape-from-silhouette, photometric methods that estimate surface normals by analyzing shading information have the advantage of reconstructing fine details. Photometric stereo (PS) traditionally inputs images from a fixed viewpoint with at least three known light sources (Woodham 1980), and it has extensions for uncalibrated (i.e., unknown lighting conditions) settings (Mo *et al.* 2018). PS is also used for plant shape reconstruction, for example, in Arabidopsis plants viewed from above (Bernotas *et al.* 2019) and venation patterns of leaves (Zhang *et al.* 2018). Although reducing the number of required lighting conditions of PS is fundamentally ill-posed (called the shape-from-shading problem for a single-view setting), Uto *et al.* (2020) proposed a photometric method for leaf angle estimation under sunlight by introducing domain-specific priors.

### Plant/tree structure from 3D shape

**Fig. 5.**
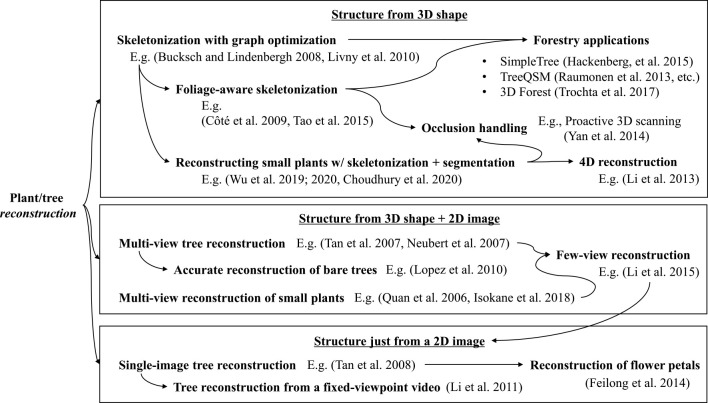
Developmental trends in the estimation of reconstructed plant/tree structure. Top: Estimation methods using 3D shapes. Middle: Methods jointly using 3D shapes and 2D images. Bottom: Methods just using 2D images.

This section discusses the methods used to extract the structure of plants and trees from reconstructed 3D shapes, such as point clouds. We deal with the shapes that are acquired by any method, for example, 3D laser scanners, RGB-D sensors (e.g., Microsoft Kinect), and multiview stereo. Meanwhile, some methods estimating structure information from 3D shapes alone implicitly assume the point clouds captured by 3D laser scanners, whose accuracy is relatively high. The methods unifying 2D and 3D information to estimate plant structure—often used for multiview or single-image input—have been discussed later. Fig. 5 summarizes the taxonomy of tree/plant structure estimations from 3D shape and/or 2D images.

Naïve 3D shape representations such as point clouds do not have structural information. In addition, some 3D models may not be complete; for example, due to occlusions during the capture. Some plant *modeling* methods that fit the branches with the designated 3D volumes (e.g., Runions *et al.* 2005, 2007) create a branching structure; however, they do not ensure that the resultant structure accurately represents the actual plant.

#### Skeletonization + graph optimization 

To reconstruct faithful skeletons from 3D shapes, skeletonization methods (Bucksch 2014) have been studied in the CG research field, which mainly inputs *bare* trees or plants with narrow leaves such as maize. Note that, although the context is beyond plant shoot reconstruction, the skeleton structure of plant roots is often reconstructed using similar approaches (Bucksch *et al.* 2014). An early method of tackling the 3D to skeletal branch structure was developed in the late 1990s (Verroust and Lazarus 1999), although it was not limited to plant reconstruction. This method connects the neighborhood points and optimizes the branch structures by solving the shortest path problem. Bucksch and Lindenbergh (2008) constructed an octree-graph for efficient and robust skeletonization, which was later extended for partially occluded point cloud input (Bucksch *et al.* 2010). Tagliasacchi *et al.* (2009) treated thin objects as compositions of partial cylinders and developed a robust method to skeletonize partially missing point clouds.

As an important breakthrough, Livny *et al.* (2010) optimized the graph structure (called the branch structure graph; BSG) on a given 3D point cloud. Given an initial BSG using a graph-based method, for example, solving the shortest path problem optimizes the branch path and thickness regarding the fitness to the point cloud and the smoothness of branches. This method creates a plausible branch structure with minimal user interaction (i.e., just pointing at the root position) for real-world point clouds, including multiple trees. The optimization-based method is further improved; for example, some methods (Aiteanu and Klein 2014, Wang *et al.* 2014b) deal well with the varying point density captured from one side of the tree. The quality measures for these tree-skeleton reconstruction methods are provided in (Boudon *et al.* 2014).

#### Forestry applications of skeletonization methods 

Skeletonization-based methods for tree reconstruction from 3D point clouds captured by laser scanners are actively applied in forestry. The resultant models are usually called quantitative structure models (QSMs) in forestry research. SimpleTree/SimpleForest (Hackenberg *et al.* 2015a) is an interactive tool for modeling tree structures from point clouds based on forestry studies (Hackenberg *et al.* 2014, 2015b). TreeQSM (Åkerblom *et al.* 2018, Disney *et al.* 2018, Markku *et al.* 2015, Raumonen *et al.* 2013, 2015) is also used for many practical applications, such as species-specific analysis (Zhang *et al.* 2020), species recognition (Åkerblom *et al.* 2017), and estimation of above-ground biomass (Calders *et al.* 2015, Gonzalez de Tanago *et al.* 2018). 3D Forest (Trochta *et al.* 2017) is yet another popular tool for QSM reconstruction, which includes sophisticated functionalities such as the segmentation of individual trees (Krůček *et al.* 2020). Owing to the availability of sophisticated tools (including other implementations such as PypeTree (Delagrange *et al.* 2014) and AdTree (Du *et al.* 2019)), these techniques are widely used for forest-level reconstruction and practical use in forest inventories (Liang *et al.* 2016, 2018).

#### Foliage-aware skeletonization 

A major drawback of skeletonization is that it is difficult to treat thick parts, such as the foliage canopy. For foliage trees, Xu *et al.* (2007) first reconstructed the main (visible) branches and detected the rough leaf positions. They then reconstructed the invisible branches that roughly fit the leaf volumes. Similarly, Côté *et al.* (2009) segment the point clouds into woody and foliage parts based on intensity values (i.e., laser reflectance). Approximations of foliage areas as the composition of *lobes* (Livny *et al.* 2011) or volumetric models (Xie *et al.* 2018) are also used for foliage-aware reconstruction. When the laser reflectance is not accessible, the segmentation of the foliaged and woody parts is nontrivial. A few studies have addressed this problem of segmenting foliage versus woody parts based on shape information (Digumarti *et al.* 2018, Tao *et al.* 2015a, 2015b). However, even if accurate segmentation is given, it is unrealistic to achieve a physically accurate reconstruction of occluded parts only from the 3D shapes of the visible part. Nevertheless, recent attempts have analyzed the detailed crown shape to recover invisible branches (Zhang *et al.* 2014b).

#### Reconstructing small plants with skeletonization and segmentation 

For relatively small plants, it is sometimes possible to capture detailed point clouds with relatively mild occlusion, such as by capturing from multiple viewpoints so that they minimize the occluded part. Many methods on this line have been proposed using multiview reconstruction or 3D scans. Meanwhile, for small plants, naïve skeletonization approaches are often insufficient because of the availability of wide leaves. Therefore, region-segmentation techniques are often used for 3D point clouds (Nguyen and Le 2013, Xie *et al.* 2020). For example, the segmentation of individual plants from point clouds of corn crops was discussed in (Zermas *et al.* 2018). Stem-leaf segmentation from a point cloud was also developed in Miao *et al.* (2021), Sodhi *et al.* (2017) by solving the classification of stems vs. leaves. The segmentation of the voxel-based reconstruction was considered in das Choudhury *et al.* (2020). In addition, from a photogrammetry-based 3D point cloud, segmentation of organs (leaves, branches, and fruit) of grapevine (Dey *et al.* 2012) and segmentation of leaf instances (Li *et al.* 2020a, Santos *et al.* 2014) are discussed. A combination of leaf segmentation and skeletonization is often applied to plants with relatively narrow leaves, for example, to extract the structure of maize (Wu *et al.* 2019, 2020) or Arabidopsis viewed from the side (Chaudhury and Godin 2020), cotton plant (Sun *et al.* 2021), and sorghum (Gaillard *et al.* 2020).

#### 4D (time-series 3D) reconstruction 

Beyond 3D reconstruction, part segmentation for each leaf and branch is a helpful cue for temporal tracking. Li *et al.* (2013) developed an accurate method of reconstructing the 4D (i.e., time-series + 3D) plant structure, including branches, leaves, and buds, from time-series 3D point clouds. Similar concepts were used for plant phenotyping (Chebrolu *et al.* 2021, Magistri *et al.* 2020), leaf tracking (Gelard *et al.* 2018), visualization (Golla *et al.* 2020), and analysis of blooming flowers (Zheng *et al.* 2017). A recent study provided a time-series point cloud dataset for the 4D phenotyping of maize and tomato (Schunck *et al.* 2021).

#### Occlusion handling 

Some methods attempt to resolve invisible parts, mainly by targeting small plants or specific parts of plants. A phenotyping system of grape clusters proposes the use of 3D scans (Schöler and Steinhage 2015), which involves prior knowledge of the spherical shape of grape grains to mitigate the impact of occlusions. In addition, the use of X-ray CT is a possible method for resolving the occlusions of small plants, which has been used for flower reconstruction (Ijiri *et al.* 2014). Another unique way of (physically) treating occlusion is letting the users sweep the occluders away. *Proactive* 3D scanning (Yan *et al.* 2014b) for example, tracks the movement of swept objects by the 3D scanning system and reconstructs the occluded part. Yet another way is to physically break down the whole plant (Yin *et al.* 2016), as discussed at the beginning of this section.

### Plant/tree structure from 2D images + 3D shape

Image-based 3D reconstruction methods, such as photogrammetry and volume-based reconstruction, rely on multiview images. Using 2D cues on each image is a powerful method because of the many resources for 2D image analysis resulting from CV-related studies. Although the context is beyond 3D reconstruction, in the plant phenotyping field, there have been many attempts using 2D image analysis, such as leaf counting and segmentation (Minervini *et al.* 2016, Scharr *et al.* 2016) and organ detection (David *et al.* 2020), where large datasets were constructed for both tasks, such as the CVPPP Dataset (Minervini *et al.* 2016) and the Global Wheat Head Dataset (David *et al.* 2020, 2021). In fact, an early attempt of multiview 3D reconstruction of botanical trees (Shlyakhter *et al.* 2001) unified the 2D and 3D cues. It used a shape-from-silhouette for 3D shape acquisition and inferred an L-system-based structure from the reconstructed model by extracting the candidates of branch tip points on 2D silhouettes. The points were then back-projected onto the 3D space to determine 3D branch tips.

#### Multiview tree reconstruction 

In 2007, two famous methods for multiview tree reconstruction were proposed in the popular CG conference SIGGRAPH. A paper entitled *image-based tree modeling* (Tan *et al.* 2007) utilized 2D-3D joint information. From the multiview image input, they first created SfM-based sparse point clouds. This method used 2D image segmentation between the foliage and woody parts to determine the visible branch part. Visible branches traced the branches from the root 3D point using the cost function defined with both 3D distance and 2D image gradient so that the branches did not cross the object edges on images. Later, they created hidden small branches so they would fit the canopy volumes. However, the method by Neubert *et al.* (2007) takes the opposite strategy: generating the structure by gradually unifying small branches. They created a vector (attractor) field on each 2D image based on the direction of the root and the density of foliage/branches. They then produced a number of particles in 3D space and moved them to trace the created vector field. The resultant paths of the particles form a tree skeleton.

More recently, Guo *et al.* (2020b) proposed a fine method to reconstruct foliaged trees using depth images reconstructed from multiview image input as guidance. Because the reconstruction of foliage is challenging, Bradley *et al.* (2013) focused on this specific topic, yielding a method to estimate detailed (per-leaf) reconstruction of dense foliage using template shapes of leaves, while this direction is improved in Chaurasia and Beardsley (2017) to use parametric leaf models. However, similar to the discussions for 3D-shape-based methods, it is still unrealistic to estimate physically correct branching patterns for foliaged trees due to the inevitable heavy occlusions.

Previous approaches have achieved accurate reconstruction of branch structures using multiview images of bare (i.e., unfoliaged) trees. Lopez *et al.* (2010) first estimated the branch skeletons in each 2D image and then integrated them into a 3D space. Zhang *et al.* (2015) used the tracking of image features between neighboring multiview images and used them for branch skeleton extraction. The method proposed by Zamuda *et al.* (2011) shares a similar concept but uses an evolutionary algorithm to optimize the branching parameters that fit the given multiview images.

#### Multiview reconstruction of small plants 

For small plants, Quan *et al.* (2006) proposed an interactive method involving leaf segmentation jointly using 2D and 3D features. The method also recovers the occluded branch structure through user interactions. To achieve automatic reconstruction of leafy plants with inevitable occlusions, Isokane *et al.* (2018) proposed the use of deep learning for 2D image processing. By inputting multiview images, they first convert the leafy plant images to 2D branch probabilities using an image-to-image translation network (Isola *et al.* 2016). They then aggregated the 2D probabilities onto 3D space and generated the branch structure using the particle-based method (Neubert *et al.* 2007). Doi *et al.* (2020) proposed a method to reconstruct leaf instances from multiview images and applied them to soybeans. They first perform instance segmentation on each 2D image and then estimate the multiview correspondences to yield 3D leaf instances. The use of instance segmentation to recover 3D structures was also proposed in Santos *et al.* (2020), where they tracked the instances over a video captured by a moving camera.

#### Few-view reconstruction 

Decreasing the required number of viewpoints is a practical research direction for multiview reconstruction. From the two images, Teng *et al.* (2007) extracted skeletons of bare trees and then unified the 2D skeletons in 3D space. Using RGB-D images, which contain both RGB color information and depth information, is an instant way of decreasing the input images because it is relatively straightforward to bring 2D segmentation to 3D point clouds. Using a single RGB-D image is often discussed as a robotics application because RGB-D cameras are often mounted on mobile robots and used for 3D leaf segmentation tasks (Alenya *et al.* 2011). For robotic pruning applications, a single RGB-D image is used to segment and reconstruct tomatoes (Li *et al.* 2015) or to find pruning points from dormant apple trees (Akbar *et al.* 2016). RGB-D captured from above is beneficial for specific applications, such as reconstructing flower petals from a single RGB-D image considering occlusion (Zhang *et al.* 2014a).

### Plant/tree structure from a single 2D image

The 3D reconstruction of plants from a single RGB image is fundamentally ill-posed. Moreover, it will be beneficial because of its extreme applicability. Tan *et al.* (2008) proposed a method for tree reconstruction from a single image. The algorithm resembles their previous method for multiview input (Tan *et al.* 2007), where both methods trace the branch paths from the root point and generate 3D branches using predefined rules. These approaches have been further extended to include single-image-based methods (Argudo *et al.* 2016) to provide a more plausible appearance. Guénard *et al.* (2013) used the analysis-by-synthesis strategy; they first reconstructed the initial skeletons using skeletonization and then refined the underlying parameters of branching systems to fit the observed silhouette. A recent method by Liu *et al.* (2021) uses a generative adversarial network (GAN) to create 3D tree models from a single image. As an extension of single-image tree modeling methods, a video captured from a fixed viewpoint is used to reconstruct 3D tree animation (Li *et al.* 2011). The focus of these single-image-based modeling approaches is to generate 3D tree models that provide nice-looking trees. It is fundamentally difficult to reconstruct physically correct 3D shapes, unless there is a strong prior knowledge, such as flower petals (Yan *et al.* 2014a).

## Conclusions and future directions

This paper summarized past and current trends in plant modeling and reconstruction methods, which are categorized into 1) creation of virtual (non-existent) plants and trees (referred to as *modeling* in this paper) and 2) modeling from real-world plants and trees (i.e., *reconstruction*). The representation of the shape and structure of plants and trees was also reviewed. A number of methods have been proposed so far but there is significant room for improvement. This review concludes with some open problems resulting from the limitations of existing works, as well as promising future directions for this research field.

### Occlusion-aware structure reconstruction

A major limitation of existing reconstruction methods is the difficulty of accurately recovering hidden structures, although there have been a few attempts to recover the occluded part using deep learning (Isokane *et al.* 2018). Occlusion handling is essential in practice because the foliage of plants and trees naturally involves heavy occlusions.

### High-throughput 3D reconstruction

The entire pipeline of the 3D reconstruction process (capturing and reconstructing the target object) is time-consuming. In particular, image-based methods, such as photogrammetry, often require special equipment for capturing a single plant, which restricts its use in field phenotyping. There is a strong demand for detailed reconstruction (i.e., structural recovery per single leaf and branchlet) with higher throughput (e.g., using drone photographs), as some researchers are actively pursuing this direction (Guo *et al.* 2020c).

### 4D reconstruction

An important application of plant structure reconstruction is growth monitoring and analysis. Although there have been attempts to recover 4D structures of plants (Li *et al.* 2013), these methods require relatively complex equipment for a single target plant. 4D reconstruction from a simple input (e.g., by a few cameras) is beneficial.

### Analyzing plant functionality using reconstructed models

For plant phenotyping studies, the primary goal of plant modeling and reconstruction is to analyze plant functionality, which is an essential part of cultivation and breeding. Although the current main topic of plant phenotyping is reconstructing or extracting the characteristics of plant phenotypes, as reconstruction techniques will grow, the extracted detailed traits will be actively used for plant science and breeding.

## Author Contribution Statement

FO wrote the manuscript.

## Figures and Tables

**Table 1.** Common methods for 3D shape reconstruction along with their rough classification and characteristics. Active settings rely on external light sources whose positions and directions are known. Geometric methods use triangulation or 3D ray intersections, while photometric methods analyze the irradiance values captured by cameras

Setting	Approach	Method	Input	Assumption	Output	Scale
Passive	Geometric	(Two-view) stereo	Two images with disparity	Known camera poses (position/orientation)	Distance to each pixel (i.e., depth image)	Yes
		Structure-from-motion (SfM)	Multi-view images	Unknown camera poses	Camera pose + sparse 3D points	No
		Multi-view stereo (MVS)	Multi-view images	Known camera poses	Dense 3D point cloud or 3D mesh	No
		- Shape from silhouette - Space carving - Computed tomography (CT)	Multi-view images	Known camera pose	3D voxel occupancy or density	Yes
	Learning (or optimization)	Single-image 3D reconstruction	A single image	Using a pre-trained neural network or a parametric shape model on the specific domain	Depth image or surface normal (+ reflectance, structure, etc., depending on methods)	Yes/No
Active	Direct	- Time-of-flight (ToF) - 3D laser scanners/LiDAR	Light (temporal) pattern + receptor		Distance to each point (usually as a 3D point cloud or depth image)	Yes
	Geometric	Active stereo (structured light)	Light (spatial) pattern (e.g., by projector) + camera	Known relative pose between projector & camera	Distance to each point/pixel (usually as depth image)	Yes
	Photometric	Photometric stereo (PS)^*a*^	Images (fixed viewpoint) with different light source	Known/unknown light position (depending on methods)	Surface normal (+ reflectance and/or camera pose, depending on methods)	No
		Shape from shading^*b*^	A single image	Known light source + surface reflectance (and additional constraints)	Surface normal	No

^*a*^ Passive setting of PS is possible using *uncalibrated* methods captured under unknown lighting positions. ^*b*^ Active but casual setting using the sunlight (and its direction acquired by latitude/longitude and time) is a possible extension.
